# Successful mechanical thrombectomy with an aspiration catheter for fenestrated basilar artery occlusion guided by preoperative basi-parallel anatomical scanning

**DOI:** 10.1016/j.radcr.2024.09.044

**Published:** 2024-09-19

**Authors:** Masanori Sato, Yosuke Nishimuta, Hiroshi Hosoyama, Yuya Shigehatake, Fumio Miyashita, Hiroshi Tokimura, Ryosuke Hanaya

**Affiliations:** a Department of Neurosurgery, Graduate School of Medical and Dental Sciences, Kagoshima University, 8-35-1 Sakuragaoka, Kagoshima-shi, Kagoshima, 890-8520, Japan; b Division of Neurosurgery, Kagoshima City Hospital, 37-1 Uearata-cho, Kagoshima-shi, Kagoshima, 890-8760, Japan; c Division of Neurology, Kagoshima City Hospital, 37-1 Uearata-cho, Kagoshima-shi, Kagoshima, 890-8760, Japan

**Keywords:** Aspiration, Basi-parallel anatomical scanning, Cerebrovascular disease, Endovascular treatment, Fenestrated basilar artery occlusion, Ischemic stroke

## Abstract

Basilar artery (BA) fenestration and its occlusion are relatively rare conditions. Mechanical thrombectomy for fenestrated BA occlusion has a high risk of complications. One limb occlusion or partial occlusion of fenestration mimics arterial stenosis or dissection. We present the case of a 75-year-old woman who presented with slight dysarthria, which subsequently worsened. Magnetic resonance imaging, magnetic resonance angiography, and basi-parallel anatomical scanning (BPAS) revealed BA fenestration and occlusion of the larger limb of the fenestrated BA, for which we performed thrombectomy with aspiration and achieved Thrombolysis in Cerebral Infarction Grade 3 flow restoration without procedure-related complications. If BA occlusion occurs at a site where a thrombus does not normally occur, confirming the anatomy of the BA before thrombectomy is desirable. As we obtained information on BA fenestration and occluded limb diameter using preoperative BPAS, we were able to safely achieve effective recanalization by guiding a relatively large-diameter aspiration catheter to the thrombus coaxially with a micro-guidewire and microcatheter.

## Introduction

Basilar artery (BA) fenestration and its occlusion are relatively rare conditions. BA fenestration occurs due to the incomplete fusion of primitive longitudinal neural arteries [1]. The diameters of 2 arterial limbs are usually smaller than that of the parent artery [2]. Inappropriately sized devices can lead to severe complications. Errors or lack of image evaluation raises the risk of vascular damage and hemorrhage. One limb occlusion or partial occlusion of fenestration mimics arterial stenosis or dissection [3,4]. Balloon angioplasty can result in serious complications. In case of misdiagnosis with dissection, appropriate intervention may not be performed. Safer and effective thrombectomy can be performed if the fenestration is detected beforehand. To safely achieve effective recanalization for a fenestrated BA occlusion, important clinical issues to consider are which imaging modality to use to detect a fenestration and which device to use. Herein, we report a case of BA occlusion in which BA fenestration was detected preoperatively using basi-parallel anatomical scanning (BPAS), and effective recanalization with aspiration was safely achieved.

## Case report

**Fig. 1:**
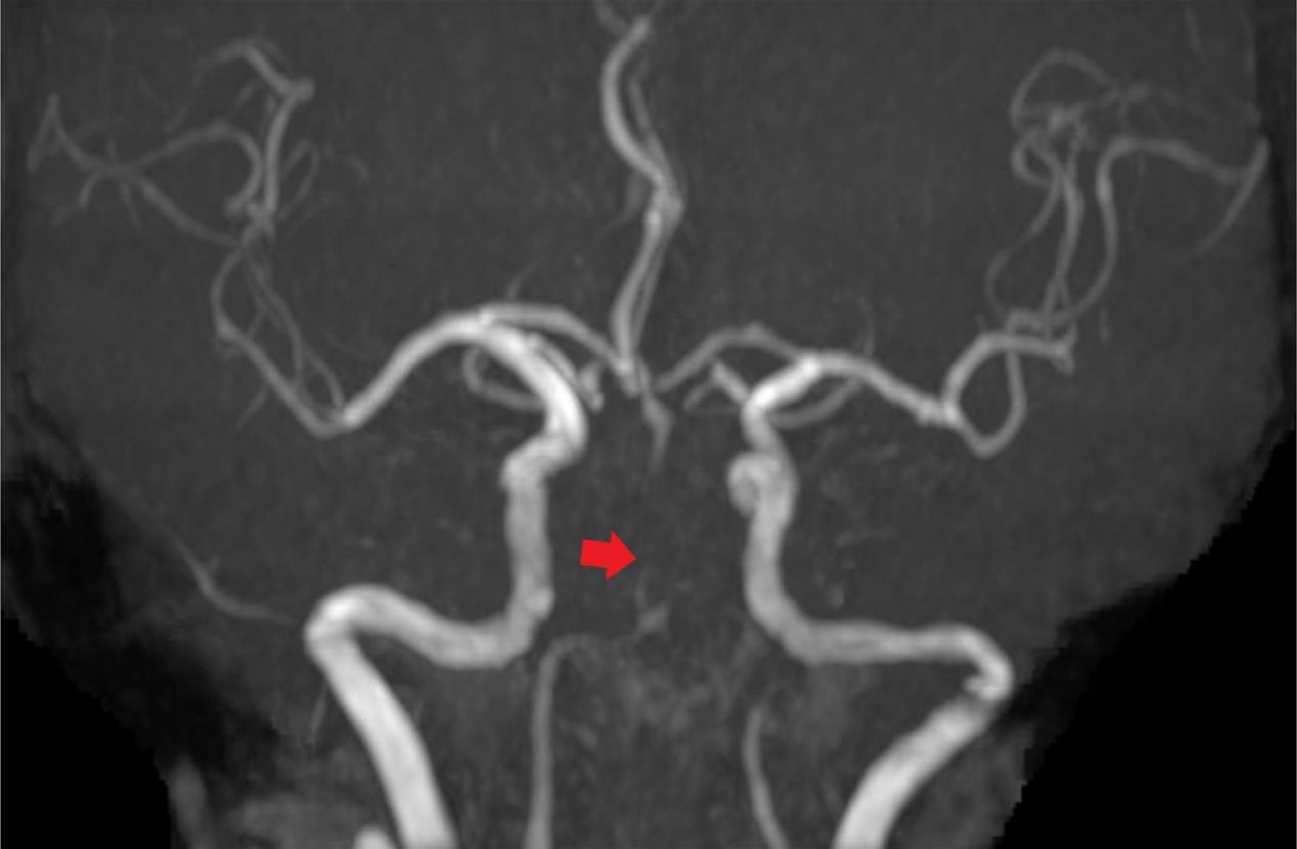
Magnetic resonance angiography (MRA) at admission. Although the basilar artery is poorly visualized on MRA, it is visible on the periphery. Severe stenosis of the basilar artery was suspected.

**Fig. 2:**
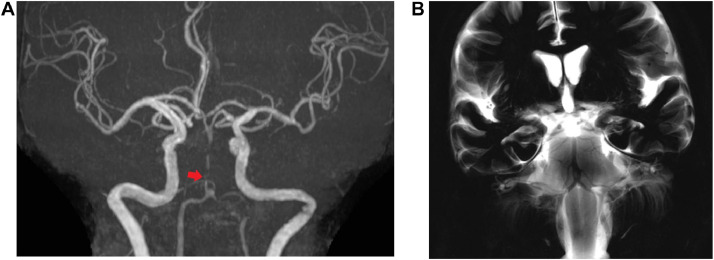
Magnetic resonance angiography (MRA) 8 hours after admission. (A) MRA indicates severe stenosis of the basilar artery, similar to the initial one. (B) Basi-parallel anatomical scanning reveals basilar artery fenestration and that the diameter of the left side major thick lumen is approximately the same as the proximal or distal side of the fenestration of the basilar artery. The left anterior inferior cerebellar artery originates from the occluded major limb.

**Fig. 3:**
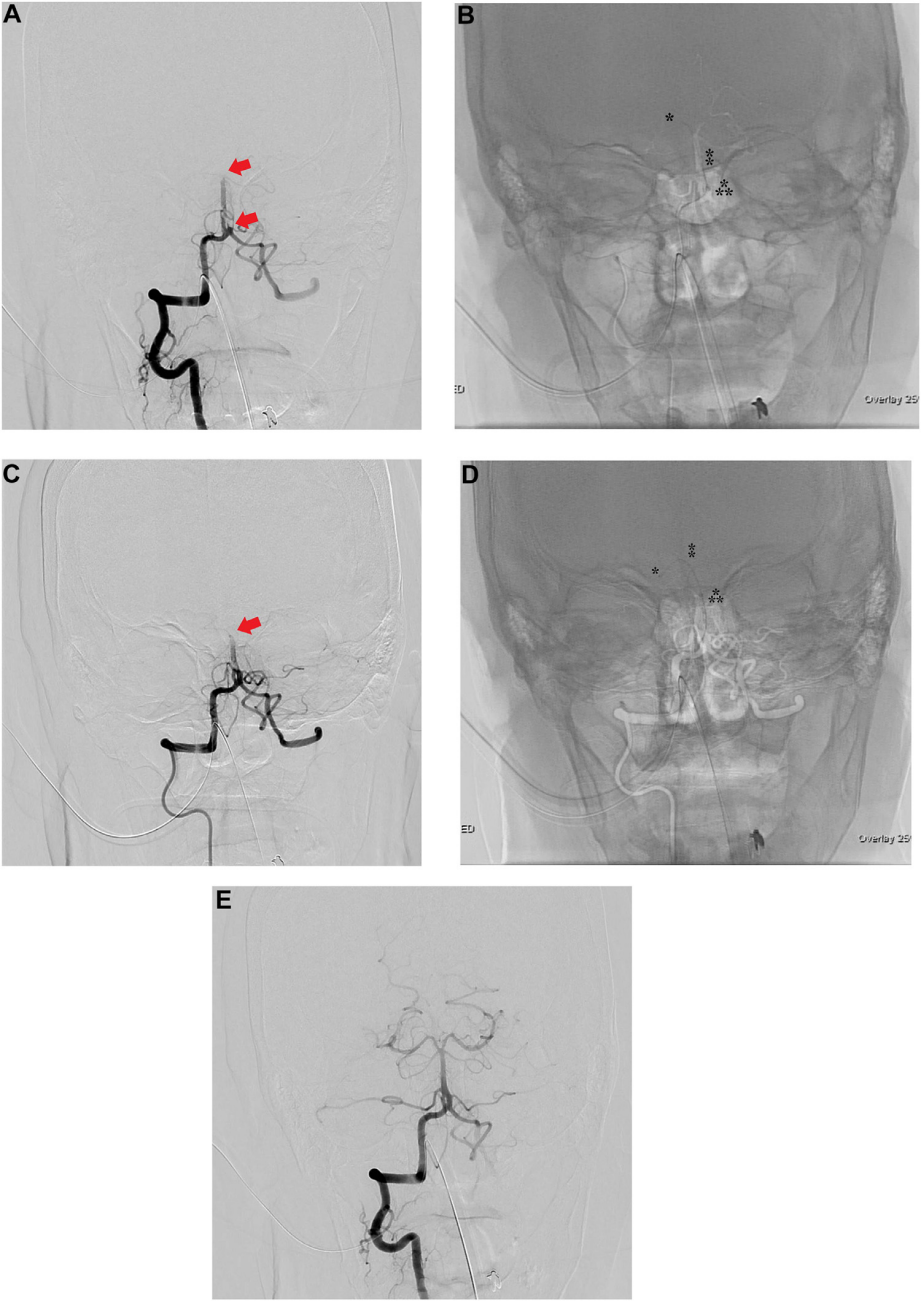
Digital subtraction angiography and thrombectomy. (A) Under local anesthesia, a 6-Fr guided catheter (FUBUKI; ASAHI INTECC, Aichi, Japan) is positioned at the right vertebral artery (VA) via the right brachial artery. Preoperative right VA angiography reveals thrombus occlusion of the major limb of the fenestration and basilar artery (BA) distal. (B) The microcatheter (⁑) (Phenom 21; Medtronic, California, USA) is placed distal to the fenestration of the BA through the major limb along with a 0.014-inch microguidewire (*) (Synchro SELECT soft; Stryker, Michigan, USA). A 5-Fr aspiration catheter (⁂) (SOFIAFLOW; TERUMO, Tokyo, Japan) is navigated to the major limb along with a microcatheter, where it is brought into contact with the thrombus, and the thrombus is aspirated. (C) Although the thrombus of the major limb was removed, and the fenestration of the BA was recanalized, the thrombus at the distal BA remained. (D) The microcatheter (⁑) is placed in the right posterior cerebral artery through the major limb along with a microguidewire (*). The aspiration catheter (⁂) is navigated to the distal BA along with the microcatheter, where it is brought into contact with the thrombus, and the thrombus is aspirated. (E) The thrombus of the BA distal is removed, and the BA is completely recanalized.

A 75-year-old woman without any specific past medical history associated with brain infarction, who was not on any medication, noticed sudden-onset dizziness, nausea, and incomplete right hemiplegia. Approximately 2 hours after onset, the patient was transported to our hospital. Upon arrival, the patient was conscious and had no neurological symptoms other than slight dysarthria (NIHSS score 1/42). An electrocardiogram showed atrial fibrillation. No acute cerebral infarction was detected on the diffusion-weighted image (DWI). Magnetic resonance angiography (MRA) showed severe stenosis of the proximal to mid-BA, suggesting atherosclerotic stenosis (Fig. 1). Treatment with oral aspirin (100 mg per day) and continuous heparin venous infusion (10,000 units per day) was initiated. Approximately 8 hours after the start of the medication, the patient became unable to speak, reaching close to complete right hemiplegia (NIHSS score 10/42). Upon reexamination of the MRI findings, the DWI revealed an acute cerebral infarction in the ventral pons (posterior circulation - Acute Stroke Prognosis Early CT score 8/10). Although there was no significant change in the MRA findings, we performed BPAS imaging to evaluate the anatomy of BA, considering the possibility of dissection. MRA and BPAS revealed occlusion of the major limb of BA fenestration (Fig. 2). As atrial fibrillation was observed at admission, a cardio-embolic stroke was suspected. We decided to perform endovascular revascularization due to the onset of acute cerebral infarction and deterioration of neurological symptoms. The major limb of the fenestration and distal BA were thrombosed. A 5-Fr aspiration catheter was navigated to the thrombus in the BA fenestration and distal BA along with a 0.021-inch microcatheter and 0.014-inch microguidewire (Fig. 3). After thrombectomy with aspiration, Thrombolysis in Cerebral Infarction Grade 3 flow restoration was achieved without procedure-related complications. Based on the digital subtraction angiography findings during thrombectomy, worsening symptoms and pons infarction was speculated to have occurred because a secondary thrombus was formed due to stump blood flow in the distal part of fenestration, which subsequently occluded the distal BA.

Her neurological symptoms generally improved except slight dysarthria and right clumsy hand syndrome after revascularization (NIHSS score 1/42). MRI after thrombectomy detected no new infarction or complication. We determined that the patient had experienced an embolic cerebral infarction due to paroxysmal atrial fibrillation, and we therefore initiated treatment with postoperative oral edoxaban (60 mg/day). The patient was transferred for rehabilitation 12 days after admission. Ninety days after thrombectomy, the patient had completely recovered, with an NIHSS score of 0/42 and a modified Rankin scale of 0.

## Discussion

In this case, we successfully performed thrombectomy with aspiration of fenestrated BA occlusion. Recanalization was achieved approximately 16 hours after the initial occlusion, resulting in a good outcome. Similarly, Tortora et al. reported a case of recanalization of BA occlusion with a good outcome more than 12 hours after symptom onset [5]. A recent study suggested that good collateral flow and distal BA occlusion, rather than recanalization time from symptom onset, were associated with good clinical outcomes [6]. In our case, we believe that the symptoms worsened owing to distal BA occlusion caused by a secondary thrombus formation due to stump blood flow. Since the posterior communicating arteries were well developed, the collateral flow was considered sufficient until symptoms worsened.

BA fenestration is relatively rare [7]. Embryologically, both limbs of the unfused BA may have perforated brainstem arteries or branches on their respective sides. Small perforating arteries originate from both the major and minor limbs of BA fenestration [8]. In our case, the left anterior inferior cerebellar artery branched from the occluded major limb. If both limbs are occluded, there is a risk of perforator or branch area infarction; therefore, recanalization of both limbs has been speculated to be preferable.

The thrombectomy device is selected based on the diameter of the parent artery; therefore, if fenestration is present, the device will be oversized. If the presence of fenestration is not known before thrombectomy, both stent retriever and aspiration catheter can cause vascular injury. Reportedly, no significant differences exist between thrombo-aspiration, stent-retrieval thrombectomy, and the combined technique in terms of their safety and efficacy [9]. Nevertheless, a meta-analysis on BA occlusion also suggested that first-line aspiration is associated with a higher recanalization rate, lower complication risk, and shorter procedure time than the use of a stent retriever [10]. For device comparison, Table 1 shows cases of thrombectomy of the middle cerebral artery (MCA) and BA occlusion with fenestration. While there have been many reports of thrombectomy with fenestration in recent years, which is thought to be attributed to the development and performance improvements of devices, only 1 case with Merci retriever (Concentric Medical Inc., California, USA) was associated with a bleeding complication [11].

**Table 1:** Overview of the cases of thrombectomy with fenestration.

Author [reference]	Sex/Age (years)	Fenestration	Occlusion	Etiology	Was fenestration known before thrombectomy?	Device and (Technique)	Pass	TICI	Complication
Seo [14]	M/44	Right M1 mid	Proximal to fenestration	ESUS	N	GDC 360 soft coil (mechanical clot disruption)	NA	NA (remaining thrombus at lower limb)	N
Namioka [11]	F/76	Left M1 mid	Proximal to fenestration	Af	N	Merci retriever V 2.5 mm soft	1	3	Hemorrhage
Nishimuta [19]	M/65	Right M1 proximal	Proximal to fenestration	Af	N	4 mm 20 mm Solitaire+5 MAX ACE (CT)	1	2b	N
Meinel [15]	M/76	BA mid	Left major limb	ESUS	Y	Solitaire (SR)	NA	3	N
Liao [20]	M/65	Left M1 distal	Both limbs subocclusion	A-to-A embolism due to left ICA stenosis	N	4 mm 20 mm Solitaire+Navien 058 (CT: SWIM)	1	3	N
Miyoshi [16]	M/71	Right M1 distal	Proximal to fenestration	Emboli formed by turbulent flow or flow stagnation around fenestration	Y	5MAX ACE68 and 3MAX (AS)	1	3	N
Hosokawa [21]	M/73	Right M1 proximal	Upper limb	Af	N	4 mm 40 mm Solitaire (SR)	1	3	N
Zhang [3]	M/51	BA mid	Distal of fenestration	Distal stenosis of fenestration	Y	4 mm 20 mm Solitaire (SR)	1	3	N
	F/65	Left M1 proximal	Proximal to fenestration	Heart valve replacement, Af	N	1:Revive SE, 2: 6 mm 30 mm Solitaire (SR)	2	3	N
Nakadate [4]	M/60s	Left M1 proximal	Proximal to fenestration	Af	N	5MAX ACE68 (AS)	2	3	N
	M/70s	Left M1 mid	Upper limb	NA	Y	5MAX ACE68 and 3MAX (AS: 2-stage aspiration)	1	NA	Distal migration of the thrombus
Kuwahara [12]	F/49	Right M1 mid	Proximal to fenestration	ESUS	N	1-2pass: Salva 71, 3-4 pass: Saliva 71+4 mm 40 mm Solitaire, 5 pass: Saliva 71+6 mm 40 mm Solitaire (AS and CT: ASAP)	5	3	N
Our case	F/75	BA proximal	Left major limb+distal to fenestration	Af, thrombus formed stump blood flow	Y	5Fr SOFIAFLOW (AS)	2	3	N

Af, atrial fibrillation; AS, aspiration; A-to-A, artery to artery; ASAP, a stent-retrieving into an aspiration catheter; BA, basilar artery; CT, combined technique; ESUS, embolic stroke of undetermined source; F, female; ICA, internal carotid artery; M, male; N, Fenestration was not known before thrombectomy or no complication; NA, not available; SR, stent retriever; SWIM, stent retriever partially retracted with intermediate catheter for mechanical thrombectomy; TICI, Thrombolysis in Cerebral Infarction; Y, Fenestration was known before thrombectomy

In 7 cases, a stent retriever was used, including 1 case in which aspiration was changed to the combined technique. Three cases used the combined technique. In all cases, except for one in which aspiration was changed to the combined technique, effective recanalization was achieved with a minimal number of passes. A stent retriever has a possibility of sticking at the fenestration. However, there were no complications in any of the cases using the stent retriever. Solitaire (Medtronic, Minnesota, USA) was used in all cases, including one that was switched from Revive SE (Codman, Massachusetts, USA). Kuwahara et al. speculated that the sheet-like overlapping structure of Solitaire allowed it to be adjusted to a narrow limb [12]. Zhang et al. further reported the characteristics of DSA after deployment of a stent [3], showing that detection of fenestration that was not preoperatively detected may be possible during thrombectomy. If the vessel diameter matches the stent specifications, 1 device may be able to deal with occlusion distal to the fenestration, or both limbs of fenestration with different vessel diameters.

Of the 4 reported cases, including ours, in which aspiration was used, effective recanalization was achieved with a minimal number of passes. There was 1 case of distal migration of the thrombus [4]. In case 1 of the study by Nakadate et al. [4], as well as the case reported by Kuwahara et al. [12], both patients had occlusions proximal to the fenestration, and aspiration at the point proximal to the fenestration revealed fenestration that was not visible before thrombectomy. The aspiration technique may also be able to identify fenestration during the procedure. For occlusion distal to the fenestration, the aspiration catheter size must be selected according to the diameter of the major limb to reach the thrombus. There is a possibility that its effectiveness will decrease. Two-stage aspiration, as shown in case 2 of Nakadate et al. [4], may be effective in this situation. Unlike a stent retriever, 2 aspiration catheters may be required for occlusions with fenestration with different diameters on both sides for effective recanalization.

Stent retrievers require the navigation of a microcatheter and microguidewire. They have a smaller diameter than aspiration catheters. Therefore, the risk of vascular injury with fenestration appears to be lower than that with aspiration, especially when fenestration status is unknown. Stent retrievers are more likely to cause bleeding complications than aspiration in BA occlusion [10], which is thought to be due to perforation of perforator branches by the microcatheter in invisible areas [13]. BA fenestration may increase the risk because the vascular course is different from normal. Because BA without fenestration is relatively straight, aspiration catheters may not require navigation with a microcatheter and microguidewire. In fenestrated BA, navigation may be necessary, but the microguidewire and microcatheter may be sufficient to the proximal end of the obstruction. Therefore, even if fenestration is present, the risk of aspiration bleeding may be lower than with stent retrievers.

Seo et al. previously reported on the prediction of vessel anomaly due to unusual resistance while advancing a microcatheter across the occluded segment [14]. However, it may be difficult to identify whether it is a hard thrombus, vessel anomaly, or other condition. Thrombectomy can cause severe complications if it is performed for a thrombus at or distal to the fenestration without awareness that the fenestration exists. There were 5 cases, including ours, in which fenestration was detected before the thrombectomy. In the case reported by Meiel et al. [15] and in case 2 of Nakadate et al. [4], fenestration was detected due to obstruction of 1 limb. In the case reported by Miyoshi et al. [16], fenestration was detected due to partial occlusion of the main artery before fenestration. Case 1 reported by Zhang et al. [3] had an obstruction of the distal BA rather than fenestration and partial recanalization after passing of the microcatheter revealed fenestration, and balloon angioplasty was considered before fenestration was recognized. In the case of an uncommon BA embolic occlusion site with an underlying embolic disease [17], it is recommended to confirm the anatomy of the BA before thrombectomy, considering the possibility of fenestration. If MRI is used, imaging BPAS should be considered. In recent years, computed tomography (CT) perfusion may be the first modality. Fenestration may be confirmed by retrograde blood flow in the late phase of CT angiography. In our case, preoperative BPAS revealed not only the fenestration, but also the anatomy of the vessels distal to it. Kuribara et al. reported that obtaining MCA information with preoperative 3-dimensional fast imaging employing steady-state acquisition for thrombectomy in the MCA region can increase the treatment efficiency and reduce hemorrhagic complications [18]. In our case, preoperative BPAS revealed BA fenestration, while the diameter of the occluded limb was similar to that of the proximal or distal BA. Therefore, we safely achieved effective recanalization by coaxially guiding a relatively large-diameter aspiration catheter to the thrombus using a micro-guidewire and microcatheter.

Using an aspiration catheter, we achieved safe and effective recanalization of fenestrated BA occlusion through preoperative BPAS. This experience suggests that effective recanalization may be safely achieved during thrombectomy for patients with BA fenestration, to obtain preoperative vascular information. However, this was a single-case study. In thrombectomy for fenestrated BA occlusion, prediction of fenestration and selection of appropriate preoperative evaluation are debatable. Additionally, whether the use of an aspiration catheter, stent retriever, or the combined technique is more effective or safer remains controversial. Therefore, further cases and randomized controlled trials are necessary.

## Conclusion

As we obtained information on fenestrated BA occlusion using preoperative BPAS, we were able to safely achieve effective recanalization by aspiration. If the BA occlusion occurs at a site where a thrombus does not normally occur, confirming the anatomy of the BA before thrombectomy is desirable. Additionally, clinicians must consider that 1 limb occlusion or partial occlusion of fenestrated BA mimics arterial stenosis or dissection.

## Patient consent

Written informed consent was obtained from the patient for the publication of this case report and any accompanying images.

## References

[1] Sogawa Keiji, Kikuchi Yoichi, O'uchi Toshihiro, Tanaka Michihiro, Inoue Tomio (2013). Fenestrations of the basilar artery demonstrated on magnetic resonance angiograms: an analysis of 212 cases. Interv Neuroradiol.

[2] van Rooij S B T, Bechan R S, Peluso J P, Sluzewski M, van Rooij W J (2015). Fenestrations of intracranial arteries. AJNR Am J Neuroradiol.

[3] Zhang Xiaoxi, Hua Weilong, Zhang Lei, Zhang Yongxin, Zhang Yongwei, Liu Jianmin (2022). Case report: fenestration embedded in large vessel occlusion at non-branching site: a catastrophic trap for mechanical thrombectomy. Front Surg.

[4] Nakadate Masashi, Kondo Ryushi, Ishihara Shoichiro, Uemiya Nahoko, Kakehi Yoshiaki, Hidaka Yukihiro (2024). Two cases of mechanical thrombectomy in patients with fenestration of the M1 Segment of the middle cerebral artery. NMC Case Rep J.

[5] Tortora Mario, Tortora Fabio, Guida Amedeo, Buono Giuseppe, Marseglia Mariano, Tarantino Margherita (2022). Basilar artery occlusion (BAO) revascularization after more than 12 hours from the onset of symptoms with excellent outcome: report of a case. Radiol Case Rep.

[6] Kwak Hyo Sung, Park Jung Soo (2020). Mechanical thrombectomy in basilar artery occlusion: clinical outcomes related to posterior circulation collateral score. Stroke.

[7] Sogawa Keiji, Kikuchi Yoichi, O'uchi Toshihiro, Tanaka Michihiro, Inoue Tomio (2013). Fenestrations of the basilar artery demonstrated on magnetic resonance angiograms: an analysis of 212 cases. Interv Neuroradiol.

[8] Im So-Hyang, Kwon Bae Ju, Jung Cheolkyu, Seo Hyung Suk, Lee Dong Hoon, Han Moon Hee (2007). Coil embolization of “kissing aneurysms” associated with distal basilar artery fenestration. Clin Neurol Neurosurg.

[9] Bai Xuesong, Zhang Xiao, Gong Haozhi, Wang Tao, Wang Xue, Wang Wenjiao (2023). Different types of percutaneous endovascular interventions for acute ischemic stroke. Cochrane Database Syst Rev.

[10] Zhang Juan, Wang Yongbin, Ju Yanmei, Jiang Hongxin (2023). Endovascular treatment of acute basilar artery occlusion: a systematic review and meta-analysis of first-line stent retriever versus direct aspiration. Brain Behav.

[11] Namioka Ai, Kadoyama Shigeru, Kato Koichi, Konishi Takanori, Nakagawa Masanori, Ujiie Hiroshi (2012). Fenestration of the middle cerebral artery detected after mechanical thrombectomy 10 using a Merci retriever system. Jpn J Stroke.

[12] Kuwahara Kiyonori, Nakahara Ichiro, Matsumoto Shoji, Suyama Yoshio, Morioka Jun, Hasebe Akiko (2024). Mechanical thrombectomy for occlusion of the fenestrated middle cerebral artery M1 segment: a case report and review of the literature. Radiol Case Rep.

[13] Choi Jin Wook, Han Miran, Park Jung Hyun, Jung Woo Sang (2020). Effect of manual aspiration thrombectomy using large-bore aspiration catheter for acute basilar artery occlusion: comparison with a stent retriever system. BMC Neurol.

[14] Seo Byung-Sun, Lee Yoon-Soo, Lee Jeong-Ho, Lee Hyuk-Gee, Ryu Kee-Young, Kang Dong-Gee (2012). Mechanical thrombolysis using coil in acute occlusion of fenestrate m1 segment. J Cerebrovasc Endovasc Neurosurg.

[15] Meinel Thomas Raphael, Pult Frauke, Gralla Jan, Arnold Marcel, Bassetti Claudio, Jung Simon (2019). Successful endovascular recanalization of a partially occluded basilar artery fenestration. Interv Neuroradiol.

[16] Miyoshi Hiroyuki, Watanabe Yosuke, Kajiwara Yoshinori, Takechi Akihiko (2021). A case of mechanical thrombectomy for occluded middle cerebral artery fenestration. Jpn J Stroke.

[17] Mutke Matthias A, Potreck Arne, Schmitt Niclas, Seker Fatih, Ringleb Peter A, Nagel Simon (2023). Exact basilar artery occlusion location indicates stroke etiology and recanalization success in patients eligible for endovascular stroke treatment. Clin Neuroradiol.

[18] Kuribara Tomoyoshi, Haraguchi Koichi, Ogane Kazumi, Matsuura Nobuki, Ito Takeo (2015). 3D-FIESTA magnetic resonance angiography fusion imaging of distal segment of occluded middle cerebral artery. Neurol Med Chir (Tokyo).

[19] Nishimuta Yosuke, Hiwatari Takaaki, Kawahara Dan, Mori Masanao, Ishii Takeshi, Yamada Masahiko (2018). Fenestration of the middle cerebral artery detected after thrombectomy: case report. Jpn J Stroke.

[20] Liao Geng, Zhang Zhenyu, Che Xiujuan, Liang Hanxiang (2020). Mechanical Thrombectomy using a stent retriever with an intermediate catheter for partially occluded middle cerebral artery fenestration. World Neurosurg.

[21] Hosokawa Makoto, Maeoka Ryosuke, Nakagawa Ichiro, Nakase Hiroyuki, Ohnishi Hideyuki (2022). Mechanical thrombectomy in acute stroke for superior limb of the fenestration of the middle cerebral artery. Radiol Case Rep.

